# Seasonal performance of a malaria rapid diagnosis test at community health clinics in a malaria-hyperendemic region of Burkina Faso

**DOI:** 10.1186/1756-3305-5-103

**Published:** 2012-05-30

**Authors:** Amidou Diarra, Issa Nébié, Alfred Tiono, Souleymane Sanon, Issiaka Soulama, Alphonse Ouédraogo, Adama Gansané, Jean B Yaro, Espérance Ouédraogo, Alfred S Traoré, Sodiomon B Sirima

**Affiliations:** 1 Centre National de Recherche et de Formation sur le Paludisme, Ouagadougou, Burkina Faso; 2 Groupe de Recherche Action en Santé, Ouagadougou, Burkina Faso; 3 UFR Sciences de la Vie et de la Terre (SVT) Université de Ouagadougou, Ouagadougou, Burkina Faso

**Keywords:** Malaria diagnosis, Transmission season, RDT, OptiMAL

## Abstract

**Backgound:**

Treatment of confirmed malaria patients with Artemisinin-based Combination Therapy (ACT) at remote areas is the goal of many anti-malaria programs. Introduction of effective and affordable malaria Rapid Diagnosis Test (RDT) in remote areas could be an alternative tool for malaria case management. This study aimed to assess performance of the OptiMAL dipstick for rapid malaria diagnosis in children under five.

**Methods:**

Malaria symptomatic and asymptomatic children were recruited in a passive manner in two community clinics (CCs). Malaria diagnosis by microscopy and RDT were performed. Performance of the tests was determined.

**Results:**

RDT showed similar ability (61.2%) to accurately diagnose malaria as microscopy (61.1%). OptiMAL showed a high level of sensitivity and specificity, compared with microscopy, during both transmission seasons (high & low), with a sensitivity of 92.9% vs. 74.9% and a specificity of 77.2% vs. 87.5%.

**Conclusion:**

By improving the performance of the test through accurate and continuous quality control of the device in the field, OptiMAL could be suitable for use at CCs for the management and control of malaria.

## Background

Despite trends toward a reduction in reported malaria cases in many epidemiological settings, malaria remains a public health problem in the majority of countries in Sub-Saharan Africa. Throughout this region, because of the unavailability of microscopy as a tool, clinical symptoms are used to diagnosis malaria when rapid diagnosis is required in order to avoid the dramatic deterioration often seen in malaria-vulnerable patients such as children under five years old. Studies of fever cases in populations [1-3]from several malaria-endemic countries, however, have found wide variations in the proportion of fevers which can truly be attributed to malaria. These countries, including the Philippines, Sri Lanka, Thailand, Mali, Chad, Tanzania, and Kenya, have shown 40–80% rates of malaria over-diagnosis, corresponding to potentially significant levels of associated economic loss. In Burkina Faso, a study of clinical examination-based malaria case definitions revealed that more than 50% of cases were not attributable to malaria alone [4]. Presumptive treatment of all fevers as malaria, previously a common practice, has therefore being questioned on economical grounds [5]. In addition, following the deployment of ACTs by the National Malaria Control Programs (NMCP); presumptive treatment is considered potentially dangerous as it might contribute to selecting for resistant *Plasmodium falciparum* strains. For these reasons, the new guidelines for malaria management recommend a mandatory laboratory test before beginning malaria treatment [6].

Microscopy has long been the method of choice for the diagnosis of many parasitic diseases, and until now it has also been the gold standard tool for malaria diagnosis. However, microscopy requires both technical skills and a power supply [7]. Solar microscopes used in the past are limited by a lack of reliable levels of sunlight, especially during the malaria high transmission season (rainy season), when most malaria cases are recorded. For efficient malaria case management at the rural level, an accurate, easy to use, and affordable malaria diagnostic tool is needed.

RDTs, introduced since the early 1990s for the detection of malaria parasites, exhibit high sensitivity and specificity compared with microscopy and Polymerase Chain Reaction [8-12]. Nevertheless, RDTs still have some limitations including the inability to detect mixed infections, all species of Plasmodium, infections at low concentrations of parasites, along with an inability to monitor response to therapy and false positives due to the presence of the malaria antigen even after successful treatment; however, their utilization in community clinics (CCs) where malaria transmission is hyper endemic and seasonal, could provide additional information for the NMCP, and would thereby serve to reduce the transmission, and consequently the burden of the disease.

Our epidemiological study aimed to assess the burden of malaria in children less than five years of age. We evaluated the sensitivity and specificity of OptiMAL compared with standard microscopy, in two CCs in the Saponé health district during malaria high and low transmission periods in 2007. OptiMAL, a dipstick test that has previously been performance tested by WHO [13] (DiaMed, Cressier, Switzerland), was used.

## Methods

### Study area

Study was conducted in the Saponé Health district in the province of Bazèga, located 50 km southwest of Ouagadougou, the capital city of Burkina Faso. The health district covers 79 villages and includes 14 CCs. The Centre National de Recherche et de Formation sur le Paludisme (CNRFP) is currently preparing this area for malaria vaccine trials. The climate of the area is characteristic of the Sudanese savannah, with a dry season from November to May (low transmission season), with a temperature range from 25°C to 30°C and a rainy season from June to October (high transmission season) with an average temperature range from 40°C to 42°C accompanied with higher humidity. Malaria transmission markedly occurs during the rainy season. Main vectors are *Anopheles gambiae,* and *A. funestus. P. falciparum* is the predominant malaria parasite, accounting for more than 95% of infections in children under five years old [14].

Usage of insecticide-treated bednets in this area is very low (1.3%), and indoor residual spraying is non-existent. Malaria control relies mainly on early diagnosis and adequate treatment of malaria episodes [14].

### Study population

Main ethnic groups in this area are Mossi and Fulani. They are mostly subsistence farmers growing millet as well as raising domestic animals. Houses are made of mud, bricks and grass or corrugated iron roofs. The study population consisted mainly of children aged 0.5 to 5 years, residing in the study villages. Study children were drawn from an epidemiological study, which aimed to assess the malaria burden (mortality and morbidity) in the health district.

### Ethical clearance

Ethical clearance was obtained from the ethics committee for biomedical research of the Ministry of Health of Burkina Faso.

### Study design and sample collection

The purpose of the study was explained to the communities by the research team. The study was carried out between January 2007 and January 2008. Informed consent was obtained from all the participants’ parents or legal guardians. The health staff of local CCs providing routine care were trained on the study procedures. Samples were collected in a passive manner as follows. Children attending the health facilities for care, presenting with fever (axillary temperature ≥ 37.5°C) or a history of fever in the previous 24 hours were enrolled. Blood was taken by finger prick for blood smears and for the OptiMAL malaria RDT, performed according to the manufacturer’s recommendations [15]. For microscopic diagnosis, blood smears were sent to the CNRFP laboratory.

To minimize observation bias, community clinic health workers performing RDT were blinded to the results of microscopic results. Technicians performing microscopic-based diagnosis were also blinded to the RDT results.

### Parasitological diagnosis by light microscope

Blood films were air-dried, thin films fixed with methanol, and the slides were stained with Giemsa 6%. Slides were read by experienced laboratory technicians who had been working mainly on malaria for many years in the CNRFP laboratory. The parasitological laboratory has been involved in the College of American Pathologist Proficiency (CAP) testing for 3 years, acheiving a good score, and most of the laboratory technicians were trained in the Malaria Diagnostic Centre of Excellence KEMRI-Walter Reed Project in Kisumu (Kenya). Briefly, slides were read according to internal SOP as follow: 100 high-power fields (HPF) were examined, and the number of malaria parasites of each species and stage recorded. The number of parasites per microliter of blood was calculated, assuming 200 white blood cells per high power field and a fixed white cell count of 8000/μl. A slide was considered negative if no parasites were found after 100 HPF were examined. Each slide was read by two independent microscopists. In the event of a discrepancy between the two readers, in terms of species, presence or absence of malaria parasites, or if parasite densities differed by more than 30%, the slide was re-examined by a third laboratory technician. Arithmetic mean of the two closest readings was used as the final value for parasite density. If there was no agreement after the third reading, the arithmetic mean of the three parasite densities was used.

### OptiMAL malaria rapid diagnostic test

OptiMAL dipstick test is an immunochromatographic assay based on the detection of Plasmodium-specific lactate dehydrogenase (pLDH) in whole blood [16]. A positive result is defined as the presence of a colored (dark purple) band on the dipstick. The test includes an internal control band (top), a pan-Plasmodium-specific band (middle), and a *P. falciparum*-specific band (bottom). pLDH is produced only by living malaria parasites and has a short half-life in the blood [17,18]. RDT was supplied regularly to the CCs and kept at +4°C to +8°C. All CCs staff members were thoroughly trained in the use of the test and interpretation of the test results before the beginning of the assessments.

One drop of whole blood was mixed with one drop of lysis buffer in well A. Lysis buffer disrupts red blood cells and releases pLDH. The specimens were then allowed to migrate to the top of the pLDH strip. After ten minutes, strips were placed for washing in well B, containing 4 drops of buffer, which clears the haemoglobin from the strip and allows appearance of bands. Interpretation of test results was performed immediately. In the pLDH assay, there are two diagnostic zones of reaction containing different antibodies. The first diagnostic zone contains a monospecific antibody that recognizes only *P. falciparum* when present. The second diagnostic zone contains a pan-specific antibody immediately above the first zone. This monoclonal antibody recognizes the pLDH of all others species. A third reaction zone containing a pan-specific monoclonal antibody is present at the top of the test strip and serves as a positive control. The test is completed in 10–15 minutes.

### Data analysis

Data double entry was done using Epi Info Software, and analysis performed using STATA, version 10 software (College Station, Texas 77845 USA) and Epi Info version 6. Sensitivity and specificity of the test were estimated using microscopy as the gold standard (Table1). Sensitivity, specificity, positive and negative predictive values were calculated as described elsewhere [19,20].

**Table 1:** Performance of the RDT compared to light microscopy

**RDT**	**Results of microscopy**		
	**Positive**	**Negative**	**Total**
Positive	1768	235	2003
Negative	205	1011	1216
Total	1973	1246	3219

The proportion of children presenting with fever or a reported history of fever with an RDT-positive test confirmed by microscopy was also calculated. Seasonal variation in the sensitivity and specificity of the test was estimated in relation to fever or history of fever. For each value, the 95% confidence interval (95%CI) (P = 0.05) was calculated. A multivariate logistic regression model was performed to examine whether sensitivity and specificity of Optimal IT varied according to season age and parasite density.

## Results

### Demographic and parasitological data

A total of 1, 242 children aged 0.5 to 5 years seeking care in two CCs were enrolled during the study period. During the study, 3, 219 visits were recorded in both clinics, with 62.4% of the visits registered during the malaria high transmission season and 37.6% during the low transmission season. The majority of children attending the health facilities, 77.4%, had axillary temperatures above 37.5°C. The sex ratio, mean age, Plasmodium indexes, parasite densities during high and low transmission seasons are summarized in Table2. Malaria-positive slides and malaria-positive RDTs were predominant during the malaria high transmission season, as compared with the low transmission season. Furthermore, *Plasmodium* indexes (*P. falciparum*, *P. ovale* and *P. malariae*) and densities of asexual stages were also high during the intense malaria transmission season. However, most of the RDT-positive samples represented *P. falciparum* nonspecific infections. Geometric mean of *P. falciparum* asexual stage density measured during the malaria high transmission season was threefold higher than that during the malaria low transmission season. *P. falciparum* was the main malaria species, with a prevalence of more than 94.2%.

**Table 2:** Demographic and parasitological data

	**Low transmission season(N = 1,210)**	**High transmission season(N = 2,009)**	**Overall (N = 3,219)**
Sex ratio	0.95	0.90	0.93
Mean age (years)	1.3	1.2	1.3
Children with fever ≥37.5 °C (%)	75.5	78.6	77.4
Children with history of fever (%)	24.5	21.4	22.6
Positive by microscopy (%)	41	73.3	61.1
Positive by RDT (%)	39.2	74.6	61.2
Positive by microscopy and negative by RDT (%)	10.7	5.2	7.3
Negative by microscopy and positive by RDT (%)	7.1	5.9	6.3
*P. falciparum* monospecific infection prevalence by microscopy (%)	37.8	71.1	58.6
RDT positive for *P. falciparum* monospecific infection prevalence (%)	28.9	66.3	52.2
*P. falciparum* gametocyte (alone) i prevalence by microscopy (%)	3.5	1.4	2.2
RDT positive for *P. falciparum* gametocyte alone (%)	0.8	0.8	0.8
*P. malariae* monospecific infection prevalence by microscopy (%)	1.1	0.4	0.7
RDT positive for *P. malariae* monospecific infection prevalence (%)	0.7	0.2	0.4
*P. ovale* monospecific infection prevalence by microscopy	0.6	0.09	0.3
RDT positive for *P. ovale* monospecific prevalence (%)	0.1	0	0.2
Mixed *Plasmodium* infections* by microscopy	3.1	2.1	2.5
RDT positive for mixed *Plasmodium* infections	2.2	2	2.1
Geometric means of *P. falciparum* parasite density	3921(3201–4804)	14615(13187–16197)	

*Mixed infections = *P. falciparum* with *P. malaria* or *P. ovale,* or *P. malariae* with *P. ovale.*

### Sensitivity and specificity of OptiMAL-IT during different malaria transmission seasons in children with fever or history of fever reported within the previous 24 hours

The overall sensitivity and specificity values of the OptiMAL-IT test during the study period were 89.6 (95%CI 88.1-90.9) and 81.1% (95%CI 78.8-83.2), respectively, with negative and positive predictive values of 88.2% (95%CI 86.7-89.6) and 83.1% (95%CI 80.9-85.2), respectively (Tables 1 and 3). During the malaria high transmission season, sensitivities were >90% in children with axillary temperatures of 37.5°C or more as well as in children with a history of fever. This dropped to less than 85% during the malaria low transmission season. The differences were statistically significant between the malaria high and low transmission seasons. In contrast, specificity was high (>85%) during the low transmission season, but decreased to less than 81% in both groups of children for the malaria high transmission season. The same patterns were observed for the predictive values (positive and negative).

**Table 3:** OptiMAL-IT performance during different malaria transmission seasons

**RDT**	**Low malaria transmission season (95% CI)**	**High malaria transmission season (95% CI)**	**P**	**Overall (95% CI)**
Sensitivity (%)	74.9(70.9-78.5)	92.9(91.5-94.1)	0.001	88.2(86.7-89.6)
Specificity (%)	87.5(84.8-89.9)	77.2(73.4-80.8)	0.001	83.1(80.9-85.2)
PPV (%)	81.8(78.0-85.2)	92(90.5-93.3)	0.001	89.6(88.1-90.9)
NPV (%)	82.3(79.3-85.0)	79.4(75.6-82.8)	0.04	81.2(78.9-83.4)

P: P value for comparison between low and high malaria transmission seasons.

PPV: Positive Predictive Value.

NPV: Negative Predictive Value.

### Sensitivity of OptiMAL-IT at different levels of peripheral *P. falciparum* parasitemia during different malaria transmission seasons

Parasitemia was categorized into six groups in order to see the variation of the sensitivity of the RDT. Sensitivity of OptiMAL-IT increased for each of the six categories during both transmission seasons (Table4). The highest sensitivities were recorded at parasite densities of >10,000 and performance dropped as parasite densities decreased. Sensitivities of >90% were reached at parasite densities of >10,000 during the low transmission season and >1,000 during the high transmission season.

**Table 4:** **Sensitivity of OptiMAL-IT at different levels of peripheral** ***P. falciparum*** **parasitemia during different malaria transmission seasons**

**Parasite threshold**	**Low malaria transmission season**		**High malaria transmission season**		
	**N**	**Sensitivity (95% CI)**	**N**	**Sensitivity(95% CI)**	**P**
[0–100]	29	10.2 (2.1-27.3)	37	45.9 (29.4-63.0)	0.001
[100–500]	76	40.7 (29.6-52.6)	91	67.0 (56.3-76.5)	0.001
[500–1,000]	41	56.0 (39.7-71.5)	66	71.2 (58.7-81.6)	0.11
[1,000-5,000]	109	82.5 (74.1-89.1)	184	90.2 (84.9-94.0)	0.05
[5,000-10,000]	37	83.7 (67.9-93.8)	114	98.2 (93.8-99.7)	0.002
> 10,000	203	97.5 (94.3-99.1)	981	98.8 (98.0-99.4)	0.24

P: P value for comparison between low and high malaria transmission seasons.

### Sensitivity and specificity of OptiMAL-IT in different age groups and during different malaria transmission seasons

The sensitivity and specificity of the OptiMAL-IT has been shown to decrease with age within each malaria transmission season. The drop in both sensitivity and specificity is markedly pronounced in older children, as compared with younger infants (Table5). During the malaria high transmission season, the sensitivity was >90% in children younger than four years old and <90% in all age groups at the malaria low transmission season. The specificities were above 80% in all age groups during the malaria low transmission season except in children > 4 years. None of the five age bands had a specificity ≥80% for the malaria high transmission season.

**Table 5:** Sensitivity and specificity of OptiMAL-IT in different age groups and during different malaria transmission seasons

**Age group (years)**	**Low malaria transmission season (95% CI)**		**High malaria transmission season (95% CI)**			
	**Sensitivity (%)**	**Specificity (%)**	**Sensitivity (%)**	**Specificity (%)**	**P**	P*
[0–1]	84.3 (75.5-90.9)	88.2 (83.8-91.8)	94.1 (91.3-96.1)	78.6 (72.5-83.9)	0.001	0.001
[1–2]	82.6 (74.0-89.4)	89.2 (83.0-93.6)	93.5 (90.5-95.8)	76.6 (68.6-83.4)	0.001	0.001
[2–3]	76.6 (68.6-83.4)	89.7 (82.7-94.5)	94.3 (91.3-96.5)	74.7 (64.5-83.2)	0.001	0.001
[3–4]	68.2 (58.4-77.0)	88.6 (79.4-94.6)	92.5 (87.9-95.7)	80.0 (66.2-89.9)	0.001	0.01
[4–5]	58.1 (46.0-69.4)	79.4 (67.8-88.2)	84.8 (77.9-90.2)	77.4 (58.9-90.4)	0.001	0.6

P: P value for comparison of sensitivity between seasons; P*: P value for comparison of specificity between malaria transmission seasons.

### Distribution of RDT false negative results by season, according to asexual parasite density

False negative rate varied similarly in both transmission seasons with more pronounced negative results with the low parasitemia (Table6).

**Table 6:** Malaria asexual parasite density and distribution of RDT false negative results, by season

**Parasite threshold (parasites/μl of blood)**	**Low malaria transmission season (N = 119)**		**High malaria transmission season (N = 100)**	
	**N**	**Frequency (%)**	**N**	**Frequency (%)**
[0–100]	26	21.8	20	20
[100–500]	45	37.8	30	30
[500–1,000]	18	15.1	19	19
[1,000-5,000]	19	15.9	18	18
[5,000-10,000[	11	09.2	13	13

## Discussion

Our results revealed that, in this rural area, children under five years old were carriers of malaria parasites at all time points of the malaria transmission season. More than half of them presenting with fever or a reported history of fever had malaria parasites in their blood, as detected either by microscopy (61.1%) or RDT (61.2%). Among these, 54.9% were RDT-positive, confirmed by microscopy. The same pattern was obtained with the OptiMAL dipstick assay, where 55.4% of all cases were diagnosed as positive by light microscopy vs. 48.8% by the OptiMAL test [21]. Thus, it appears that the performance of the OptiMAL is close to that of light microscope-based diagnosis under field conditions.

The study also showed that pLDH-based RDT performance varied seasonally in our study area. Malaria positivity rates determined by either light microscopy or RDT were high during the malaria high transmission season compared with low transmission season. Sensitivities were similarly affected by malaria transmission season, age and parasite density. Higher sensitivities were reported during the malaria high transmission season and children with high parasitemia were more likely to be positive by RDT than those with low parasitemia. This confirms previous findings from Madagascar, Kenya, and Uganda where the RDT positivities were found to be associated with high parasite load [22-26]. RDT positivity was also high in younger children, who are less immune compared with older children, confirming that sensitivity is affected by age-dependant immune status, as reported in a previous study [27].

Specificity of the RDT was not affected by age. However, it was affected by malaria transmission season; the number of false negative RDTs increased during the malaria high transmission season. These samples could have been falsely negative due to insufficient detection of low parasitemia, and pLDH levels in the samples were probably below the detection limit (20 ng/ml) of the RDT [25]. However, we cannot exclude that variability in the devices may have played a role in the performance of the test. False negative samples have been reported during both periods of transmission with higher parasitemia, and at least 40% of our subjects had parasitemia >500/μl. Though the underlying reason for this is unknown, similar findings have been reported in several earlier studies [22,28-31].

Clearly, the performance of the pLDH-based malaria RDT was greatly influenced by season. During the malaria high transmission season, the sensitivity of the test was >90% in all age groups except children above four years of age. In comparison, during the malaria low transmission season, none of the age groups had sensitivities >90%. The RDT reached the level of >90% sensitivity at parasitemia levels of >1,000/μl during the malaria high transmission season, but it required a parasitemia of >10,000/μl for the same sensitivity during the malaria low transmission season. It might be argued that the variability observed between the malaria transmissions seasons could have resulted from performance variability of the test used, especially as climatic conditions affect the stability of the devices. It has already been reported that stability is usually more problematic with pLDH-based tests than with HRP2-based tests [32].

Used in Papua New Guinea to monitor treatment outcomes, the OptiMAL dipstick showed a relatively low sensitivity (49.3%) and specificity (59.1%) [5]. The low sensitivity observed in our study may be due to the reduction in parasitemia from the treatment [22,23,33]. In a comparative study in India using OptiMAL,. Malik *et al*. obtained sensitivity and specificity values of 88.4% and 96.8%, respectively, which are comparable to those obtained in our study [34]. In Burkina Faso, OptiMAL dipsticks used with 464 hospital patients recorded sensitivity and specificity of 98.70%, and 96.25%, respectively [35]. The relatively low RDT sensitivity and specificity levels observed in our study, compared with those obtained by Valéa using OptiMAL-IT, may be due partly to the fact that, in our study setting, children were actively followed up and referred to CCs for proper management of the malaria. This may have led to a drop in malaria parasite density and, consequently, a drop in the sensitivity and specificity of the test. However we cannot also exclude that self treatment by using traditional medicines and other conventional drugs, including antimalarials, before attending the community clinic may affect the sensitivity of the test. Other significant differences between the two studies include the sample size (large in our case) and also the design of the study; in the previous study, the tests were performed by laboratory technicians posted at the hospital, whereas in our study the tests were performed by trained community clinic health workers. The community health workers may not have processed the tests as accurately as laboratory Technicians because of the increasing workload brought by this new malaria diagnosis tool. In addition, there was no specific RDT monitoring program throughout the study period. Indeed, at the peripheral level, many factors such as work load, turnover of personnel, and control of the cold chain could have led to errors during test processing that may have affected the outcome of the RDT. Many studies have shown that job aids in the form of verbal or pictorial instructions, combined with training and supervision, enhance a health worker's ability to correctly perform specific tasks [36,37]. One caveat to be heeded when implementing an alternative measure, such as RDTs, to help health workers in remote areas to diagnose and manage malaria cases is that it must first be well accepted by the community health workers. A randomized trial conducted in Burkina Faso that aimed to determine the impact of the introduction of malaria RDT on clinical decisions, failed to show good compliance by health workers [38]. Preparation of appropriate instructions and training as well as monitoring of users’ behavior and the cold chain is an essential part of rapid test implementation in remote areas.

## Conclusions

These results, combined with other advantages of the OptiMAL test, such as the rapid availability of results (10–15 minutes) and relative simplicity compared with microscopy and other confirmatory tests done elsewhere, suggest that OptiMAL can be used by relatively inexperienced people to diagnose malaria infection in rural areas. However, seasonal variation should be considered when implementing an RDT-based malaria diagnostic program. Overall proportion of fever or reported history of fever associated with positive RDT and confirmed by microscopy indicates that at least half of outpatients presenting in our CCs are infected with malaria parasites at any given time point during the malaria transmission seasons. Despite the relatively low sensitivity of the test (89.6%), the performance of the Optima IT test can be improved by putting in place an appropriate quality management system for the management of clinical malaria cases in remote areas.

## Authors' contributions

All authors read and approved the final version of the manuscript.

## Competing interests

We declare that we have seen and approved the final version. We also declare that we have no conflict of interest in connection with this paper and state that the material has not already been published and will not be submitted for publication elsewhere so long as it is under consideration by the *Journal of Parasites & Vectors.*
